# Pathogenesis of giant cell arteritis with focus on cellular populations

**DOI:** 10.3389/fmed.2022.1058600

**Published:** 2022-11-17

**Authors:** Pavlos Stamatis, Carl Turesson, Despina Michailidou, Aladdin J. Mohammad

**Affiliations:** ^1^Rheumatology, Department of Clinical Sciences, Lund University, Lund, Sweden; ^2^Rheumatology, Sunderby Hospital, Luleå, Sweden; ^3^Rheumatology, Department of Clinical Sciences Malmö, Lund University, Malmö, Sweden; ^4^Division of Rheumatology, Department of Medicine, University of Washington, Seattle, WA, United States; ^5^Department of Medicine, University of Cambridge, Cambridge, United Kingdom

**Keywords:** vasculitis pathogenesis, giant cell arteritis, innate immunity, adaptive immunity, cytokine signatures, check-point dysregulation

## Abstract

Giant cell arteritis (GCA), the most common non-infectious vasculitis, mainly affects elderly individuals. The disease usually affects the aorta and its main supra-aortic branches causing both general symptoms of inflammation and specific ischemic symptoms because of the limited blood flow due to arterial structural changes in the inflamed arteries. The pathogenesis of the GCA is complex and includes a dysregulated immune response that affects both the innate and the adaptive immunity. During the last two decades several studies have investigated interactions among antigen-presenting cells and lymphocytes, which contribute to the formation of the inflammatory infiltrate in the affected arteries. Toll-like receptor signaling and interactions through the VEGF-Notch-Jagged1 pathway are emerging as crucial events of the aberrant inflammatory response, facilitating among others the migration of inflammatory cells to the inflamed arteries and their interactions with the local stromal milieu. The increased use of checkpoint inhibitors in cancer immunotherapy and their immune-related adverse events has fed interest in the role of checkpoint dysfunction in GCA, and recent studies suggest a dysregulated check point system which is unable to suppress the inflammation in the previously immune-privileged arteries, leading to vasculitis. The role of B-cells is currently reevaluated because of new reports of considerable numbers of plasma cells in inflamed arteries as well as the formation of artery tertiary lymphoid organs. There is emerging evidence on previously less studied cell populations, such as the neutrophils, CD8+ T-cells, T regulatory cells and tissue residing memory cells as well as for stromal cells which were previously considered as innocent bystanders. The aim of this review is to summarize the evidence in the literature regarding the cell populations involved in the pathogenesis of GCA and especially in the context of an aged, immune system.

## Introduction

Giant cell arteritis (GCA) is a non-infectious vasculitis affecting medium and large size arteries, especially the aorta and its main branches (1). It is the most common vasculitis in the western world with an incidence ranging from 5.8 to 22.2 per 100 000 inhabitants aged ≥50 years (2–5). Epidemiologic studies in the northern hemisphere have shown a clear north-to-south and west-to-east gradient, with the disease affecting mostly Caucasians (2, 3, 5–7). In Europe and North America, the female to male ratio is nearly 3:1 whereas the ratio tends to be 1:1 in western Asia (6, 8). The mean age at the diagnosis is 75 years (2, 3). Headache, scalp tenderness, jaw claudication, visual symptoms, polymyalgia rheumatica and arm claudication are common symptoms of the disease. Visual loss, stroke and aortic aneurysms are among the most feared manifestations of the disease. Most patients develop concurrently constitutional symptoms as malaise, fever, anorexia and weight loss as a consequence of the uncontrolled inflammation (9, 10). Elevated inflammatory markers are present in the majority of the patients (11, 12). In the appropriate clinical context, a positive temporal artery biopsy or typical imaging findings are required for the diagnosis of GCA. Glucocorticoids (GCs) are the mainstay of the treatment and other immunosuppressive agents are administrated adjunctively to reduce the exposure to GCs (13, 14).

GCA is traditionally considered to be an immune-mediated disease where the responsible vasculitogenic antigen(s) has yet not been identified. Overexpression of MHC class II genes located in the regions between HLA-DRA and HLA-DRB1 and even the over-presentation of genes located in the HLA-DQA1 and HLA-DQA2 suggest that GCA is an antigen driven immune mediated disease (15–17). Additionally, the presence of clonally expanded T-cells in different arterial sites suggests that there is a particular response to specific epitopes (15, 18, 19).

The arterial mural layers are considered the primary fields where the events of the inflammatory cascade take place. In GCA, large and medium sized arteries with diameter ≥2,000 μm are usually affected (20). These arteries have 3 mural layers: the intima, the media, and the adventitia. The arterial wall contains endothelial cells, vascular smooth cells, elastic membranes, matrix and fibroblasts (20). In these large arteries, the necessary nutrients cannot reach all the mural layers by diffusion from the lumen, and especially the high energy-demanding media layer. A microvasculature system is necessary, to transfer all the necessary nutrients from the arterial lumen to all 3 arterial layers. This microvasculature system is also called vasa vasorum (“vessels of the vessel”). In contrast with small arteries which do not have vasa vasorum, there are resident vascular dendritic cells (vasDCs) in the interface between the media and the adventitia of large arteries (15, 20). These cells play a critical role in the pathogenesis of GCA, which involves both the innate and the adaptive immune system.

## Innate immune system in GCA

### Toll-like receptors

The Toll-like receptors (TLR) are a family of transmembrane proteins which were identified in the mid 1990's. So far 10 types of TLRs have been identified. The TLRs 1, 2, 4, 5, 6 and 10 are expressed on the cellular surface and the TLRs 3, 7, 8 and 9 are expressed in cytosolic vesicles (21). TLRs act as pattern recognition receptors (PRRs), binding pathogen-associated molecular pattern ligands (PAMPs) and damage-associated molecular pattern ligands (DAMPs) (21, 22). The PAMPs originate directly from microbial agents, whereas the DAMPs are damage products from the inflamed tissues which have been produced during the “battle” between the host's immune system and the invader (21–23). In the event of a dysregulated interaction between the immune system and the specific tissue (20), DAMPs may act as kick-starters of an inflammatory response even in the absence of infection, trauma and ischemia (24). Several studies have pinpointed dendritic cells (DCs) as key players in this dysregulated interaction between the immune system and the arterial wall (19, 20, 25). Varying combinations and patterns of TLRs may expressed in the vasDCs of different arteries and an intriguing hypothesis could be that the activation of a specific TLR pattern leads to a specific immune response in arteries sharing the same or similar TLRs pattern, offering a possible explanation for the tissue tropism in GCA (20, 26).

### Dendritic cells

DCs comprise an important link between the innate and adaptive immunity. Several studies have shown that this population of vasDCs, which is dysregulated in GCA, has a key role in the pathogenesis of the disease (19, 20). These vasDCs usually reside at the adventitia-media border and in normal arteries are tolerogenic which means that they don't have the ability to stimulate T-cells (19). A plausible hypothesis is that PAMPs and DAMPs from the main circulation (e.g., from one or more infectious agents in susceptible individuals) may reach the adventitia-media border *via* vasa vasorum (19, 26). In individuals predisposed for GCA (by TLR polymorphisms or other genetic and/or environmental factors), vasDCs may be activated by the presence of these danger signals, gaining T stimulatory capacity (15, 19, 26, 27). This activation causes the migration of these DCs into the media, where DCs produce chemotactic factors (e.g., CCL19 and CCL21) which in turn cause the migration and activation of T-cells and macrophages (19, 20). The subsequent inflammatory cascade, orchestrated mainly by Th1- and Th17-cell mediated responses, contributes to the granulomatous infiltrate seen in GCA (20, 28).

### Macrophages

The wall of normal medium-sized and large arteries is usually devoid of macrophages and T-cells (64). The macrophages are recruited to the arterial layers probably by activated vasDCs and T cells *via* vasa vasorum (15). Chemokine release from vascular smooth muscle cells (VSMCs), induced by IFN-γ, has been shown to be important for this recruitment (29). Recently, Watanabe et al. showed that monocytes from patients with GCA produce high amounts of matrix metalloproteinase 9 (MMP-9), which allows them to digest the basement membrane of vasa vasorum capillaries and enter the adventitial tissue, and thus exerting tissue-invading abilities and at the same time facilitating the invasion of other inflammatory cell populations (30).

**Figure 1:**
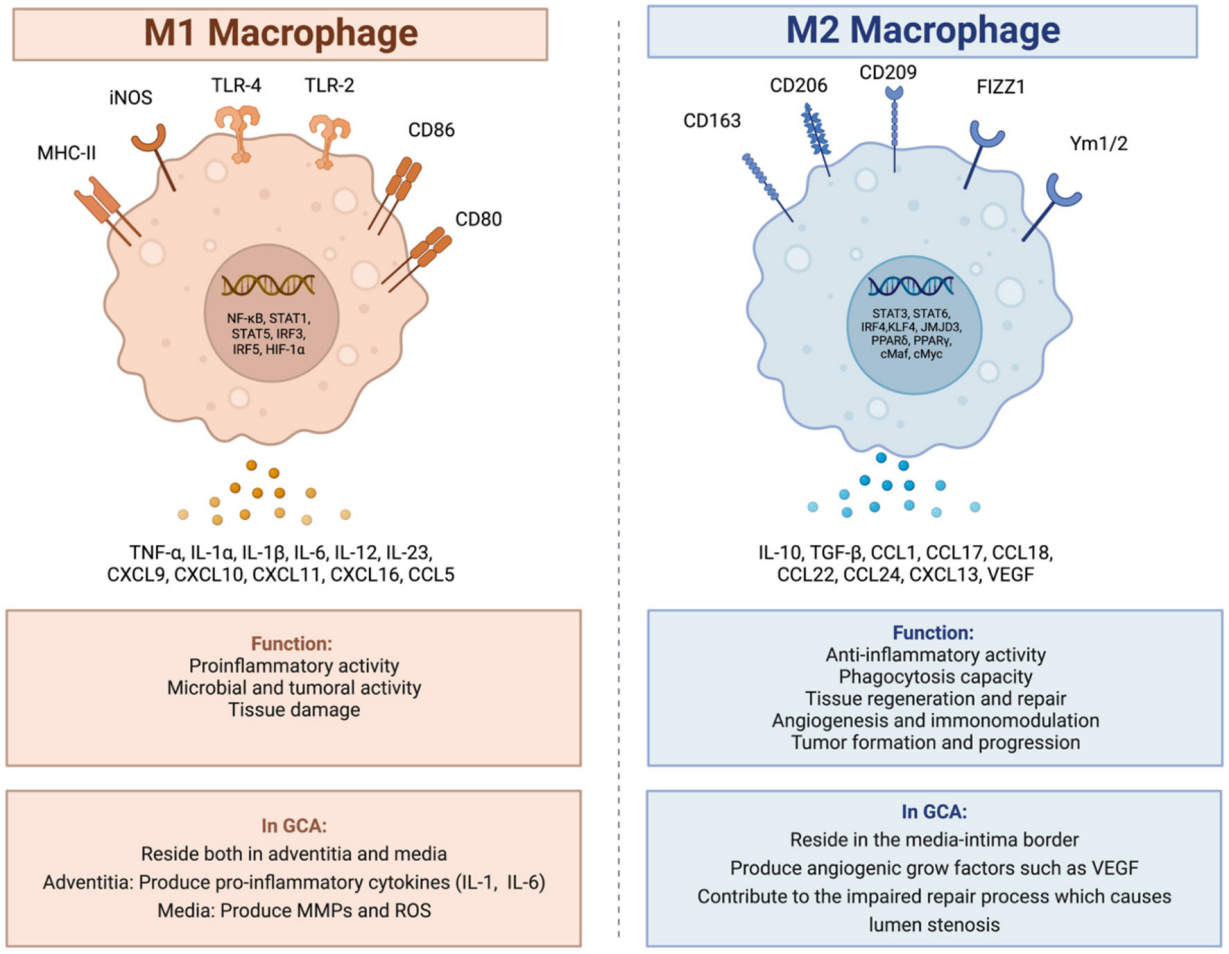
M1 and M2 macrophages, and their role in giant cell arteritis. In GCA there are two main types of macrophages. The M1 macrophages specialized in proinflammatory and tissue destructive actions and the M2 macrophages specialized in tissue repair mechanisms. Created with BioRender.com. Adapted from “Macrophage polarization: M1 and M2 subtypes”. Retrieved from https://app.Biorender.com/biorender-templates.

Among these macrophages, there are two main types, polarized in response to the microenvironment of the arterial wall: the M1 phenotype and the M2 phenotype (Figure 1) (15, 31). The M1 macrophages are specialized in proinflammatory actions whereas the M2 macrophages are more specialized in tissue-repairing mechanisms (20).

In GCA, activated M1 macrophages reside both in the adventitia and in the media. In the adventitia, the macrophages produce pro-inflammatory cytokines such as IL-1 and IL-6 contributing to the maintenance of the inflammatory response (15, 18, 31), whereas in the media, the M1 activated macrophages, produce molecules which contribute to the degradation of the arterial wall, molecules such as MMPs and reactive oxygen species (ROS) (15, 20, 32, 33).

The M2 activated macrophages reside at the media-intima border producing angiogenic growth factors such as vascular endothelial growth factor (VEGF) contributing to the morphological and structural changes of the arterial lumen with wall-thickening and stenosis as a part of a dysregulated repair process (15, 18).

The typical granulomas in GCA consist of activated T-cells, macrophages and histiocytes, usually including multinucleated giant cells (20). Multinucleated giant cells are the histopathological hallmark of giant cell arteritis found in up to 75% of the positive temporal biopsies (34–36). They are the result of fusion of activated macrophages (37). This process, and the differentiation of macrophages to effector cells in the vasculitis lesions, have been shown to be partly driven by granulocyte-macrophage colony stimulating factor (GM-CSF) and macrophage stimulating factor (M-CSF) signaling (38).

### Neutrophils

Neutrophils are crucial mediators of the innate immune response (39) and play a vital role in the pathogenesis of many autoimmune diseases *via* release of neutrophil extracellular traps (NETs) (40). Circulating immature neutrophils were recently identified in patients with GCA that extravasated into the surrounding tissues of temporal arteries and produced elevated levels of extracellular ROS leading to enhanced vascular damage (41). Some other studies have demonstrated enhanced neutrophil activation as assessed by elevated levels of N-formyl methionine (fMET) that is a potent neutrophil chemoattractant, and calprotectin in the peripheral blood of patients with GCA. Circulating fMET was capable for mounting a de novo neutrophil activation *in vitro*, in a formyl peptide receptor 1 (FPR1)-dependent manner (42, 43). Neutrophilic infiltration has also been observed at the adventitia and media of involved arteries in GCA (44, 45) with presence of NETs identified in temporal artery biopsies from patients with GCA (46).

## Adaptive immune system in GCA

### T-cells

**Figure 2:**
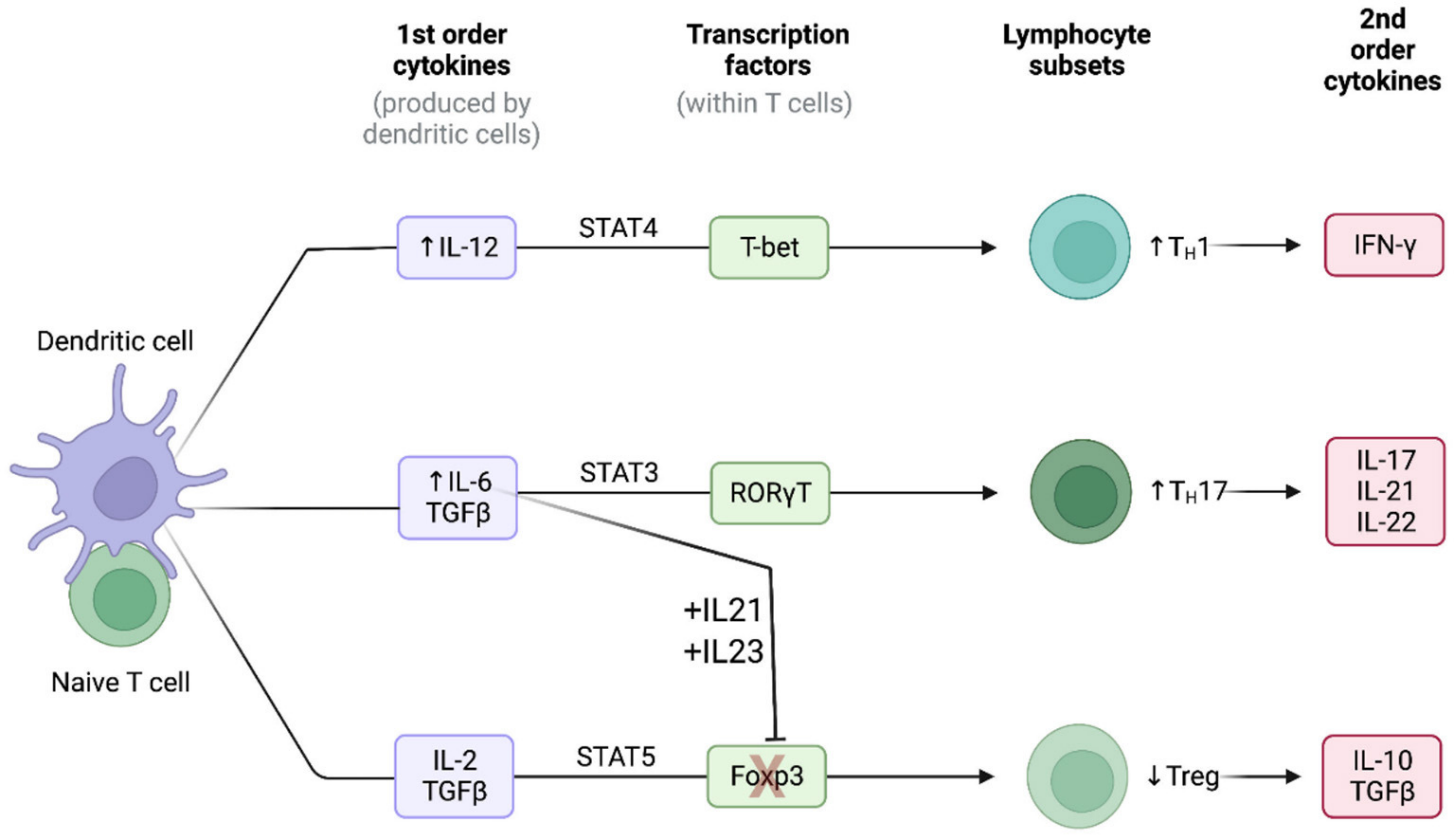
Cytokine signatures in GCA. Lineage polarization of naive T-cells in giant cell arteritis. Created with BioRender.com. Adapted from “Induction of 2nd order cytokines”. Retrieved from https://app.Biorender.com/biorender-templates.

In a manner similar to the macrophages, the medium-sized and large arteries are normally devoid of T-cells (20). In GCA, activated vasDCs, residing in the adventitia-media border near the vasa vasorum, secret chemotactic factors which attract T-cells to these arteries. Consequently, depending on the interaction between the antigen-presenting cells and T-cells, T-cells differentiate into two main T-helper (Th) lineages: the Th17 and the Th1 lineage (Figure 2) (20).

### Th17 cells

The Th17 cells constitute a key T-cell population in the pathogenesis of GCA. The frequencies of Th17 cells in the peripheral blood measured by flow cytometry have been consistently reported to be higher in patients with GCA in comparison to healthy controls (28, 47, 48). Naïve T-cells, under the stimulation of TGF-β and IL-6 or IL-21 upregulate the IL-1R and IL-23R (20). Consequently, the presence of TGF-β, IL-1β, IL-6 and IL-21 leads to the differentiation of naïve T-cells toward to the Th17 lineage (Figure 2) (49). The activated Th17 cells produce a plethora of cytokines such as IL-17, 1L-21, IL-22 and chemokine ligand 20 (CCL20) (20, 49). The production of these cytokines and chemokines contributes directly and indirectly both to the systemic manifestations of the disease and to the local arterial damage (e.g., the stimulating effect of IL-17 to macrophages). However, after treatment with GCs, both the number of Th17 cells and the concentration of Th17-related cytokines are markedly reduced in patients with GCA, both locally and in the peripheral blood of the patients (20, 28).

### Th1 cells

The Th1 cells is another sub-population of T-cells which is critically involved in the pathogenesis of GCA. Unlike Th17 cells, the number of Th1 cells and the Th1-related cytokines seem not to be affected by the treatment with GCs, as they remain elevated both in the arterial tissue and in the blood (28). The presence of IL-12 in the arterial microenvironment shifts the differentiation of naïve T-cells toward to the Th1 lineage leading to the production of the powerful inflammatory cytokine IFN-γ (Figure 2) (20, 50). IL-12 has a key role in the activation of macrophages, the production of damage molecules (MMPs, ROS etc.) and the proliferation and migration of vascular smooth muscle cells (20).

### T-regulatory cells

Abnormalities of T-regulatory cells (T-regs) also contribute to the pathogenesis of GCA. The high concentrations of IL-6, IL-21 and IL-23 in the microenvironment cause the blockage of the Forkhead box protein P3 (FOXP3), a transcriptional factor which is necessary for the differentiation of T-regs (Figure 2). Additionally, the presence of these cytokines, upregulates the transcriptional factor RORγt which stimulates the Th17 differentiation (51, 52). Consequently, the frequencies of T-regs in patients with GCA measured by flow cytometry have been reported low (47, 48). Recently, a pathogenic role of Tregs has also been proposed. Miyabe et al., demonstrated a pathogenic T-reg phenotype with impaired suppressor capacity and increased IL-17 production (53). Thus, in patients with GCA the Tregs are not only decreased in number, but their functionality is also impaired.

### CD8+ T cells

The relative low numbers of CD8+ T cells both in the peripheral blood and in affected arteries has initially implicated a limited role of CD8+ T cells in the pathogenesis of GCA (54–56). However, a defect in peripheral immunosuppressive CD8+ Tregs have been recently demonstrated in the elderly (56, 57). CD8+ T regs normally regulate the activation and proliferative expansion of CD4+ T cells. Furthermore, it is now known that CD8+ T cells are more susceptible to age-related changes and their number decrease with increased age whereas naïve CD4+ cell are more resistant to age-related changes (58, 59). Thus, CD8+ T cells may have an unexplored contribution to the induction of the disease although they are not present in great number in the inflammatory infiltrate (60).

### Tissue-resident memory cells

Tissue-resident memory T-cells (a subset of memory T-cells) appears to play a critical role in sustaining the inflammatory process. These cells stay on local tissues instead of returning to secondary lymphoid organs (61). The reason for this is to provide a rapid and effective response upon antigen re-encountering in the tissue. In GCA, it is now believed that these cells play a crucial role in the renewal and maintenance of the inflammatory infiltrate (62, 63).

### B-cells

The role of B cells in GCA is not currently understood. It is believed that the B cells do not exert a key role in the pathogenesis of GCA. Previous theories regarding specific autoantibodies (e.g., cardiolipin antibodies) which could have a role in the pathogenesis of GCA have not confirmed by other studies (64–66). However, a recent study which investigated the changes in the histopathological image between the initial biopsy and a second follow up biopsy, randomly performed 3, 6, 9 or 12 months after the initial biopsy, showed the presence of plasma cells in the inflammatory bulk. In the initial biopsies, plasma cells were recorded in the 83% of the TABs whereas the percentage was lower (40%) in the second follow up biopsy (67). In line with these observations, Ciccia et al. (25) demonstrated that in patients with GCA, and more specific in the media layer of the temporal artery, there is a unencapsulated formation consisted of B cell aggregates, follicular dendritic cells, surrounding T cells and high endothelial venules. These structures are called as artery tertiary lymphoid organs (ATLOs). The ATLOs are formed postnatally and in response to chronic inflammation. Newly formed lymphatic vessels may transfer cytokines, chemokines, antigens, PAMPs and DAMPs from the arterial microenvironment to ATLOs. ATLOs were absent in healthy controls and were present in patients with GCA independently of the presence of atherosclerosis (25, 68). Interestingly, B cell survival factors such as BAFF and APRIL were present at higher levels in patients with GCA than in healthy controls (25). These factors are produced by endothelial cells and vascular smooth muscle cells, indicating an interaction between stromal cells and immune cells (25). Recently, a study from Netherlands, following a previous study from the same group which showed low circulating B-cells in active GCA, demonstrated massive and organized B-cell infiltrates in the aorta of patients with LV-GCA (69, 70).

**Figure 3:**
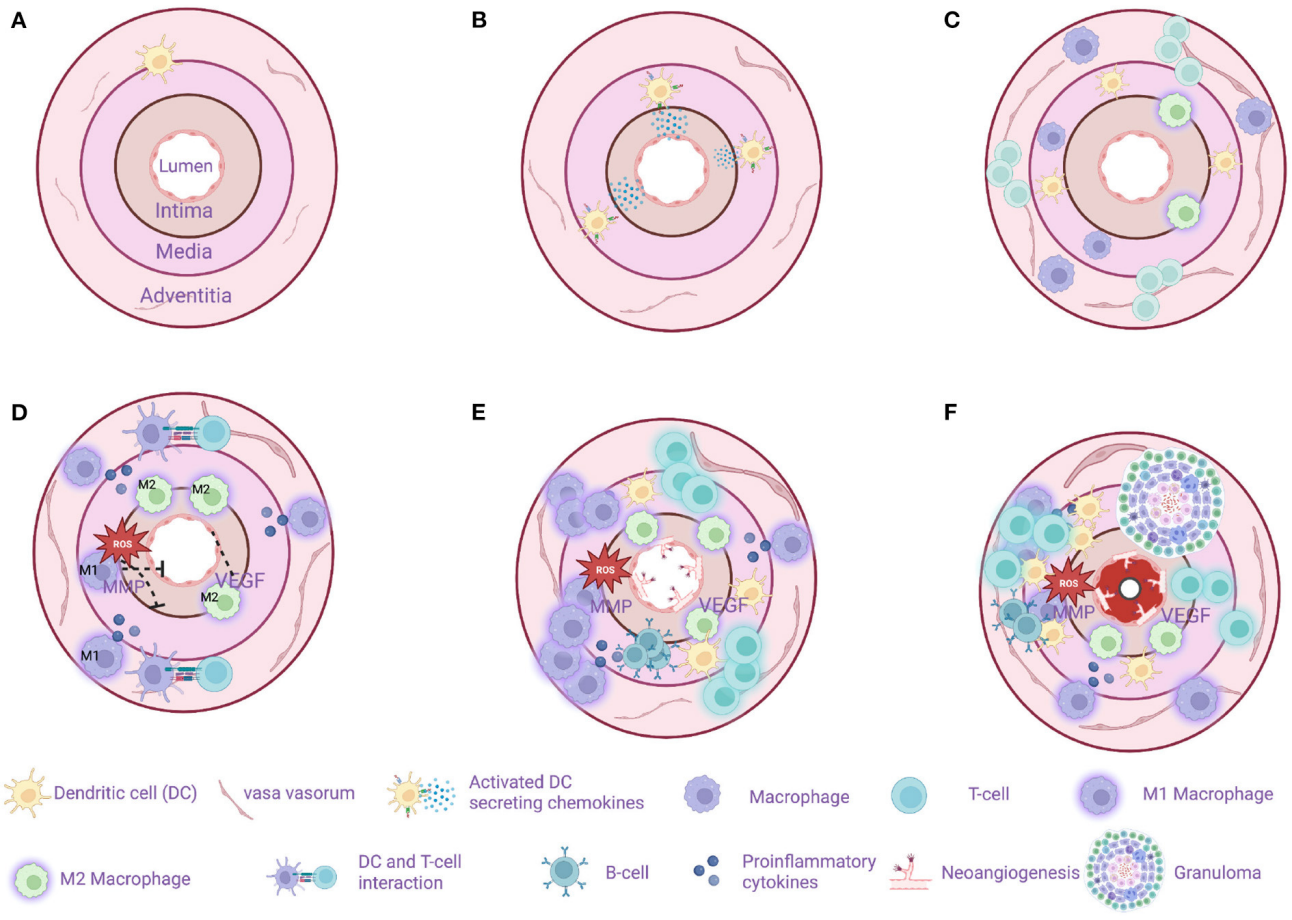
Model of sequential steps in pathogenesis of giant cell arteritis. **(A)** A healthy temporal artery is devoid of macrophages and T-cells. Vascular dendritic cells (vasDCs) are located in the adventitia-media border as a part of the immunosurveillance system. **(B)** The immunotolerance in GCA breaks and the activated vasDCs migrate to the media where they produce chemotactic factors. **(C)** Macrophages and T-cells are recruited from the main circulation probably entering the artery through the vasa vasorum. MMP-9 producing monocytes as well as the VEGF-NOTCH1-Jagged1 pathway facilitate the migration of macrophages and T-cells. **(D)** M1 macrophages located in the adventitia produce proinflammatory cytokines maintaining the inflammatory response. M1 macrophages located in the media produce tissue destructive molecules such as MMP-9 and reactive oxygen species (ROS). M2 macrophages in the intima-media border produce angiogenic growth factors such as VEGF contributing to the dysregulated repair mechanism in GCA. T cells continue to infiltrate the artery's layers with direction from adventitia to intima. **(E)** The destruction of the artery's tissue continues, T-cell inhibitory signals as the PDL1-PD1 are weakened in GCA leaving the activation of T-cell unopposed. At the same time a dysregulated repair process is in progress with excessive neo-angiogenesis and fibrosis. In some cases, artery tertiary lymphoid organs are located in the media. **(F)** The final result of the chronic inflammation is the formation of granulomas (up to 75% of the examined temporal artery biopsies) and the progressive stenosis and in some cases occlusion of the artery due to a maladaptive response to injury. Created with BioRender.com.

Figure 3 illustrates important steps in the GCA pathogenesis.

## Cytokine signatures in GCA

### The IL-6–IL-17 signature

IL-6 is a pleiotropic cytokine which can be secreted both from immune and stromal cells (endothelial cells, fibroblasts, and vascular smooth muscle cells), and is therefore an important mediator in the crosstalk between the immune system and the injured tissue (20, 71). IL-6 contributes to elevation of inflammatory markers through the activation of hepatocytes and plays an important role in the differentiation of naïve T-cells into functional lineages. More specifically, IL-6 in the presence of TGF-β steers the T cell differentiation toward the Th-17 lineage and at the same time, in synergy with IL-21 and IL-23, blocks the transcription factor FOXP3 which is essential for the differentiation of Tregs (Figure 2) (20, 71, 72). Thus, the presence of IL-6 exerts proinflammatory effects by differentiating naïve T-cells into the inflammatory Th-17 subset and restricts possible counteractions of the immune system by reducing (in absolute number and/or functionality) anti-inflammatory T-cells as T-regs (20).

The Th-17 cells produce a plethora of proinflammatory cytokines such as IL-8, IL-17, IL-21, IL-22, IL-26, CCL 20 and GM-GSF (20). The receptors of these cytokines are located both locally (e.g., IL-17 dependent activation of macrophages, endothelial cells, vascular smooth muscle cells and fibroblasts) and remotely (e.g., IL-22 mediated hepatocyte activation and production of acute phase reactants) (20, 73, 74). At early disease stages, a combination of Th-17 related cytokines is found both in the inflammatory infiltrate and in the peripheral blood. However, a previous study has demonstrated that treatment with GCs is highly effective in downregulating the Th-17-axis, by rapidly suppressing the production of IL-1, IL-6, and IL-23 cytokines, cytokines which are essential for the differentiation of Th17 cells (28). Consequently, the production of IL-17 is suppressed both in blood and in the inflamed arteries.

### The IL-12–IFN-γ signature

In GCA-affected arteries, activated vasDCs may be a source of IL-12 (20, 75). Following stimulation with IL-12, naïve CD4+ T cells undergo lineage polarization into Th1 cells, through activation of the master transcription factor T-bet and suppression of the master transcription factor GATA-3 which favors a Th2 lineage polarization (Figure 2) (72). Consequently, Th1 lineage-specific genes are expressed and Th2-related genes are suppressed. Activated Th1 cells secrete the powerful proinflammatory cytokine IFN-γ. Generally, IFN-γ is mainly produced by NK-cells, CD8+ T cells, Th1 cells, macrophages and DCs (76), and IFN-γ receptors are mostly encountered in granulocytes, monocytes and macrophages (56). IFN-γ not only enhances the ongoing inflammatory process but also intensifies the tissue injury (20). Furthermore, IFN-γ interacts with tissue stromal cells like VSMC and endothelial cells. VSMC under the influence of IFN-γ become either apoptotic or migratory, with direction toward intima, contributing to arterial luminal stenosis (20, 77). Contrary to the response of the Th-17 axis to GCs, Th-1 responses are regulated mostly by the IL-12–IFN-γ cytokines, and are therefore unaffected by standard immunosuppressive treatment, as demonstrated by studies of tissue transcripts and plasma concentrations of IFN-γ (20, 28).

It has recently been demonstrated that plasma levels of IFN-γ and other proteins related to T cell function may be elevated years before clinical disease onset (78), further underlining the importance of this pathway and suggesting that it may drive very early disease mechanisms.

## Check point dysregulation in GCA

**Figure 4:**
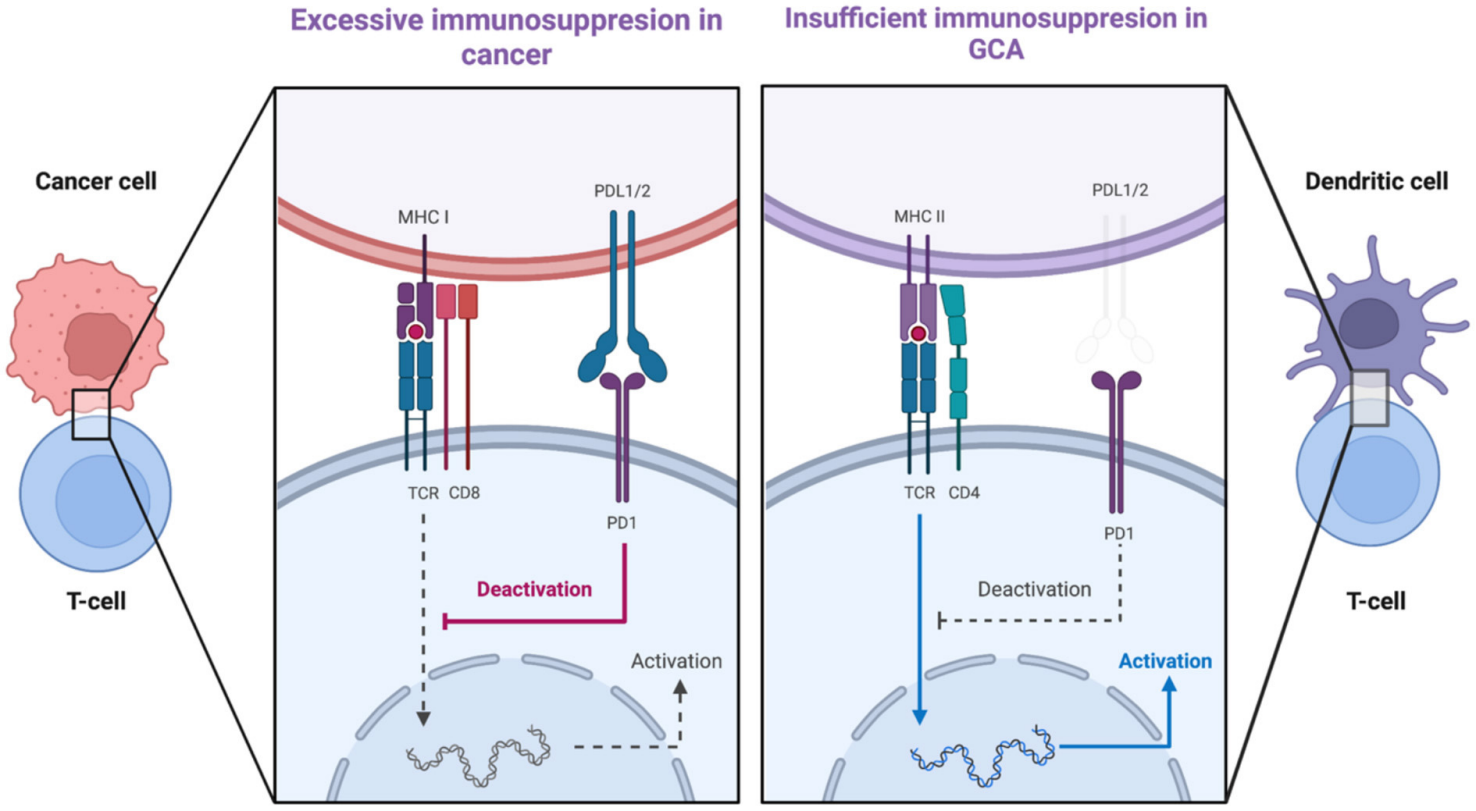
PDL1-PD1 checkpoint dysregulation in cancer and in giant cell arteritis. The expression of PDL1 on cancer cells delivers immunoinhibitory signals to T-cells and thus cancer cells evade tumor surveillance. In GCA, there is low expression of PDL1 on vascular dendritic cells whereas most T-cells arriving in the artery are PD1-positive. Created with BioRender.com. Adapted from “T-cell Deactivation vs. Activation.” Retrieved from https://app.Biorender.com/biorender-templates.

A second co-stimulatory signal (e.g., CD28/CD80-86) is required for the activation of T-cell dependent immunity when an antigen binds to a T-cell receptor (TCR) (79). This is balanced by inhibitory signals which limit T-cell activation (e.g., CTLA-4/CD80-86 and PD-1/PD-L1) (79). Malignant cells usurp the PD-1-PD-L1 pathway by expressing the immunoinhibitory ligand PD-L1. Consequently, these cancer cells deliver immunoinhibitory signals when they encounter PD-1+ T-cells and thus evade immunosurveillance (80). Therefore, the PD-1/PD-L1 checkpoint inhibition is a well-recognized target in cancer immunotherapy, providing revolutionary results by unleashing the force of T-cell-dependent immunity upon malignant cells. Interestingly, immune-related adverse events (irAEs), as the price of the uncontrolled T-cell activation, are frequent side effects of cancer immunotherapy evolving any organ or system (81). Among other irAEs, several case reports of patients developing GCA under treatment with PD-1/PD-L1 inhibitors (Nivolumab, Pembrolizumab) have been published recently (82–85). Indeed, in GCA it seems that there is a dysregulation in PD-1/PD-L1 checkpoint inhibition (86). PD-L1 is normally expressed in antigen-presenting cells, stromal cells, and tumor cells whereas PD1 is expressed in T-cells, B-cells, NK-cells, activated monocytes, and dendritic cells (80, 86). In GCA-affected arteries, there is low expression of PD-L1 transcripts with no demonstrable expression of PD-L1 in vasDCs whereas most T-cells in the granulomas were PD-1 positive (87). Therefore, a mechanism which could inhibit excessive immunity in the arteries is defective, allowing infiltrating T-cells to remain activated. Of note, in healthy arteries, there was a high expression of PD-L1 transcripts and no expression of PD-1 transcripts, a finding confirming that normal arteries are devoid of T-cells (Figure 4) (88). Furthermore, *in vivo* blocking of PD1 in an animal model of GCA [chimeras of severe combined immunodeficiency (SCID) mice with transplanted human arteries, reconstituted with peripheral blood mononuclear cells from patients with GCA], exacerbated vascular inflammation and amplified T cell cytokine production (87).

It has been suggested that expression of PD-L1, and hence the PD-L1/PD-1 regulatory pathway, is regulated by glucose metabolites (89). The positive association between mitochondrial pyruvate levels and macrophage PD-L1 expression (89) and the reduced prevalence of diabetes mellitus at GCA diagnosis (90) are compatible with a protective effect of hyperglycaemia from such dysregulation. Recently, lower fasting blood glucose levels were demonstrated in individuals subsequently diagnosed with GCA, suggesting that metabolic factors influence the risk of GCA, possibly through effects on checkpoint function (91).

## Stromal interactions in GCA

The main components of the arterial vessel wall are the endothelial cells, vascular smooth muscle cells, vasDCs, elastic membranes, matrix, and fibroblasts. Theories which consider GCA as strictly an immune-mediated disease, where an unknown vasculitogenic stimulus breaks down the self-tolerance and elicits autoimmunity, overlook two major factors: (1) the age cut-off (2) the strict tissue tropism. Recent studies have tried to shed light on the interaction between the immune system and the local stromal milieu. The emerging theme is that stromal cells or extracellular matrix components could interact with the immune system playing a key role in breaking the self-tolerance and maintaining the inflammatory process.

### Endothelial cells

Endothelial cells form a barrier between the circulating immune cells and the arterial wall. Endothelial cells have the capacity to interact with immune system by expressing various adhesion molecules, receptors, and ligands. Wen et al. demonstrated a model in which endothelial cells in vasa vasorum could induce pathogenic effector functions in CD4+ cells through VEGF-NOTCH1-Jagged1 interactions (57). An increased concentration of VEGF in the serum of patients with GCA have been demonstrated, which in turn, causes up-regulation of the Jagged1 ligand in endothelial cells of vasa vasorum (92, 93). The origin of the increased VEGF is currently unknown. Previous studies have also demonstrated that NOTCH1 is also aberrantly expressed in CD4+ cells of patients with GCA. Thus, the VEGF-NOTCH1-Jagged1 pathway facilitates the invasion of CD4+NOTCH1+ T cells into vessel wall and the polarization into Th1 and Th17 effector subsets (94, 95). Notably, a prerequisite for the invasion of T-cells in the arterial wall is that MMP-9 producing monocytes have first digested the basement membrane in order to open a way for the infiltrating T-cells (30, 63).

### Vascular smooth muscle cells

Layers of VSMC are located in the media layer of medium- and large-sized arteries. These cells express in their surface molecules which allows them to communicate with neighboring cells, e.g., NOTCH1receptors and their Jagged1 and Delta1 ligands (20). Thus, as they act as signal-transducing and signal-receiving cells, they can influence the tissue microenvironment and the communication with the immune cells which bear the same receptors and ligands (20). Furthermore, upon proper stimulation, they proliferate and migrate to the intima where they produce abnormal matrix proteins and contribute to narrowing and potentially occlusion of the lumen. In the model with implanted human arteries in SCID mice, blockade of the NOTCH signaling pathway markedly reduced the transformation of VSMC from a contractile to non-contractile phenotype in arteries with established GCA. Furthermore, the T-cell density in the infiltrate was reduced and likewise the production of IFN-γ and IL-17 (95).

Another elegant study has recently demonstrated increased concentration of endothelin 1 (ET-1) in GCA-affected arteries (96). The main source of ET1 was leukocytes and monocytes and both ET receptors were upregulated in infiltrating leukocytes, endothelial cells and VSMC. Thus, this enabled the interaction between stromal cells and infiltrating immune cells. The ET-1 mediated activation of VSMC promoted their migration from media to intima with concurrent production of MMP-2 which facilitated the fragmentation of the internal elastic lamina. The authors concluded that, beyond vascular tone regulation, the ET-1 mediated activation of VSMC plays an important role in vascular remodeling in GCA (96).

### Extracellular matrix

Aging causes changes in the structure of arterial walls. The walls of the arteries become thicker and stiffer. They also lose their elasticity and therefore are more prone to age-related comorbidities, such as hypertension (97). These structural changes also reflect changes in the composition of extracellular matrix, e.g., less elastin more collagen (97). Little is known about the potential role of extracellular matrix's components on initiating or suppressing an autoimmune response. For instance, in multiple sclerosis, galectin 1A, an endogenous glycan binding protein which is produced by stromal cells and subsequently stored in extracellular matrix, found to elicit a tolerogenic response by inducing tolerogenic DCs which produce IL-27. These DCs blunt Th1 and Th17 responses and promote the differentiation of Tregs (98).

## The role of immune aging and inflammaging

During the last decade a new research field has emerged, the field of geroscience, which investigates the link between aging and age-related chronic disease (99). Seven pillars of aging were identified: (i) adaptation to stress, (ii) epigenetics, (iii) inflammation, (iv) macromolecular damage, (v) metabolism, (vi) proteostasis, and (vii) stem cells and regeneration (99, 100). These pillars are interconnected, and interact with each other. It seems that abnormalities in each of these pillars cause inflammation which in turn affects all the other pillars, making inflammation the common denominator in the pathogenesis of age-related diseases (100). This chronic, low-grade, sterile inflammation, which increases with increasing age, is called inflammaging (100, 101).

**Figure 5:**
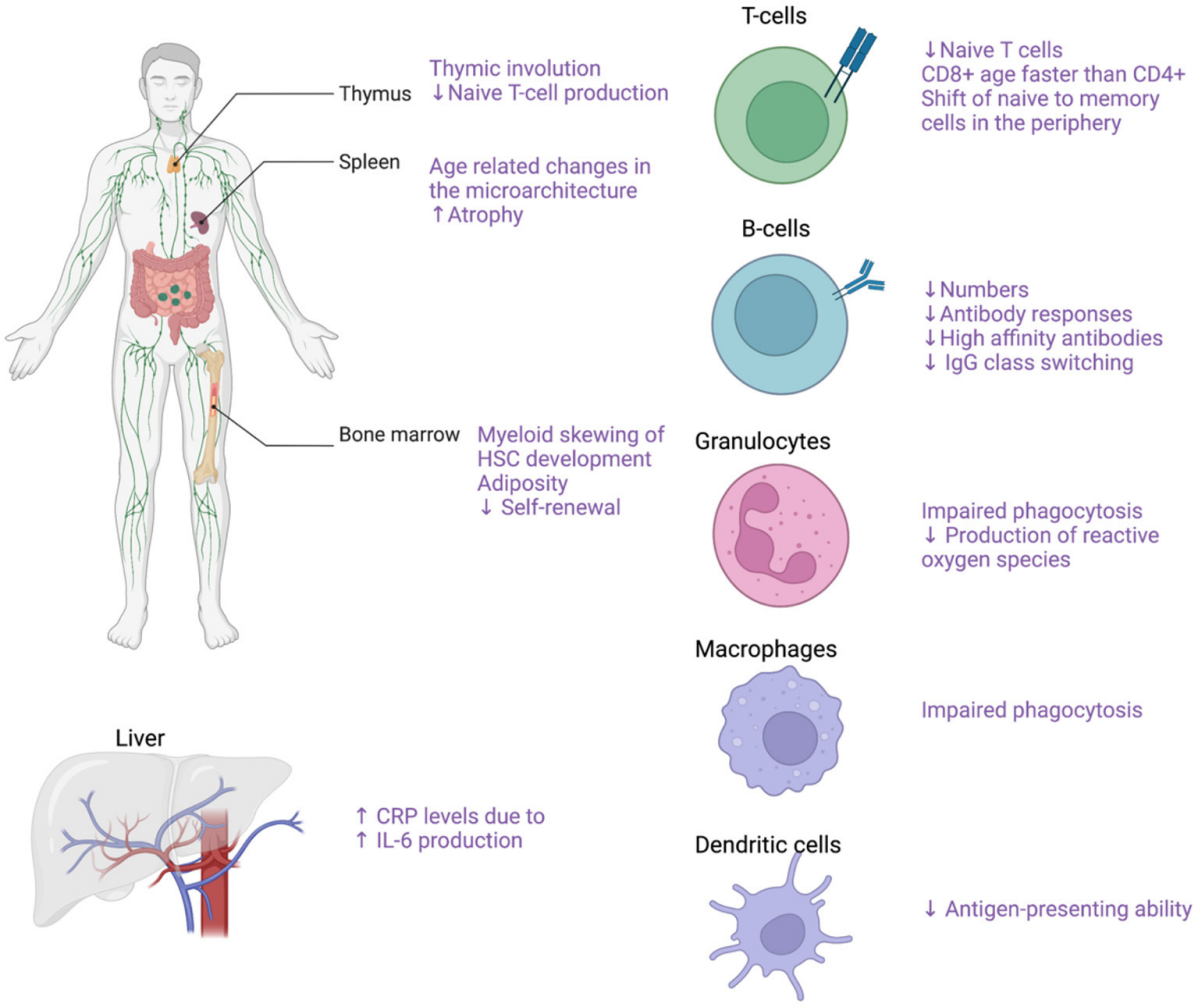
Effects of aging on the immune system. Effects of aging on the immune system with focus on the main cellular populations contributing to GCA pathogenesis. Created with BioRender.com.

The term immunosenescence describes all the age-related changes in the immune system (102). The main characteristics of immunosenescence are (1) the low-grade sterile inflammation (inflammaging), (2) the impaired wound healing, (3) the increased susceptibility to infections and cancer, (4) the lower responses to antigen stimulation (e.g. vaccinations) as well as, (5) the increased risk for autoimmune diseases (103). Both the innate and the adaptive immune system are affected by the process of aging (Figure 5). In the bone marrow, an important step toward immune aging is the myeloid skewing of hematopoietic stem cells (HSC) with decreased ability to differentiate into the lymphoid lineage (104). Changes in the microarchitecture of the spleen have also described in the elderly with increased atrophy (105). Thymus involution begins in early childhood and by the age of 75 year the thymus is mostly a fat tissue (105, 106). After the 5th decade of life and with further increased age, the number of circulating naïve T-cells, both CD8+ and CD4+ are markedly reduced although the reduction is less pronounced for the CD4+ populations (107, 108). With increasing age, there is reduced CD28 expression in both CD4+ and CD8+ lymphocytes (109–111). In individuals older than 65 years, CD4+CD28- cells represent up to 50% of the total CD4 lymphocytes whereas in young people the respective frequency ranges from 1 to 2.5% (109, 112). The reduced CD28 expression has been proposed both as a marker of normal aging and as a marker of early aging under chronic inflammatory stimulation (109, 111). Of note, these CD4+CD28- cells are potent secretors of pro-inflammatory cytokines such as IFN-γ and IL-2 (109, 113). Interestingly, increased numbers of CD4+CD28- cells have been reported both in peripheral blood and vascular lesions in patients with GCA (114). On the other hand, there is ample support for a role of CD28 co-stimulation in the pathogenesis of GCA (63), and treatment with CTLA4-Ig, which blocks CD28, has been shown to reduce the risk of relapse in patients with GCA (115). This apparent paradox may reflect co-stimulation in de novo activation of T cells that drives the disease process, leading to emergence of immunosenescent cells that retain some effector functions.

Furthermore, there is a gradual decline in the number of naïve T-cells in the periphery whereas the numbers of memory T-cells increase with age (105). The number of naïve B cells also declines with increased age as well as the quality of the humoral immune response characterized by lower antibody responses, decreased high-affinity antibodies and decreased IgG isotype class switching (105, 107, 116). The innate immune system is also profoundly affected by aging as there are several functional declines in the cellular populations comprising the innate immune system. With increasing age of the host, the granulocytes exhibit reduced functions including impaired phagocytosis and reduced production of reactive oxygen species (107, 117, 118). Macrophage phagocytosis and the ability of DCs to maturate and present antigen is also gradually impaired with aging (107). Of note, average levels of proinflammatory cytokines such as TNF-a and IL6 were reported to be increased in the elderly, leading to higher production of C-reactive protein by the liver (107, 119, 120).

## The role of infections in GCA

Cyclical fluctuations in the incidence of GCA and the granulomatous nature of the infiltrate favor theories that infection may play a role in the pathogenesis of the disease (121, 122). Epidemiological studies have shown weak to moderate associations between infections and the subsequent development of GCA (123, 124). Several studies have reported associations between antecedent infections, both viral and bacterial, and the future development of GCA (122, 125–129). However, these results were not reproducible in other, independent studies. It is doubtful whether an infectious agent could influence the immune system so profoundly and on so many levels. A more plausible hypothesis could be that an infection is the last part of the drama, where the infection causes an unpredictable and strong reaction of the immune system, because of the cumulative effect of other previous dysregulated interactions between the immune system and host tissues. On the other hand, the demonstration of elevated plasma IFN-γ levels years before GCA onset suggest that host responses to a range of different microbial pathogens may be involved in early stages of the disease process (78).

## Clinical implications

Insights on the role of cellular populations in the pathogenesis in GCA, and their variability, may help us to define clinically meaningful disease subsets. Systematic studies of tissue and blood samples may lead to identification of patients at increased risk of relapse or severe complications. Such investigations might also guide future targeted therapy.

Several targeted immunosuppressive drugs have been used as add-on to GCs, with the aim of reducing long term GC use and related toxicity (130). As expected based on the biology of IL-6, and its role in GCA, IL-6 inhibition by the anti-IL-6 receptor antibody tocilizumab is effective in GCA that has been verified by biopsy or large vessel imaging, and enables rapid GC tapering with reduced risk of relapse (13). Tocilizumab works equally well in patients with a clinical presentation dominated by cranial symptoms, and those presenting mainly with signs and symptoms of polymyalgia rheumatica. However, relapses after discontinuation of anti-IL-6 therapy do occur (131), possibly reflecting persistence of Th1 cells in chronic vascular infiltrates.

Preliminary results from a phase II randomized clinical trial indicated clinical efficacy for the IL-17A inhibitor secukinumab in patients with GCA (132). Targeting IL-17 would also be expected to affect mainly the IL-6-IL-17 pathway, potentially with greater short term anti-inflammatory effects compared to the impact on chronic aspects of the disease. These hypotheses need to be investigated in extended clinical trials.

Other agents that have been tried in the treatment of GCA include anti-CSF2 therapy using mavrilimumab (133), which blocks GM-CSF signaling, and would be expected to have an impact on giant cell formation, and JAK-inhibition [e.g. baricitinib (134)], which has a broader effect on intracellular signaling and activation of T cells and other cell populations that makes it promising as a strategy for treating GCA.

## Conclusions

GCA is characterized by an aberrant immune response involving both the innate and adaptive immunity. It is doubtful whether a single external culprit (e.g., an infectious agent) could provoke such an extensive and chronic inflammatory response. Furthermore, theories of a single external culprit fail to explain the strict tissue tropism and why GCA affects mainly the elderly. Future studies may elucidate the contribution of internal factors, such as age-related changes in cell turnover, metabolism and dealing with molecular debris, in combination with the aging immune system. Epidemiological studies have shown a lower incidence of certain types of cancer in patients with GCA after diagnosis (135, 136). The development of the disease in some susceptible individuals could therefore be an epiphenomenon of a superior tumor surveillance, as response to cancer treatment with check point inhibitors has been associated with autoimmune related adverse events, including GCA (137, 138). Innate check-point dysregulation contributing to GCA development may be influenced by metabolic factors (89). The importance of T-cell dysregulation has been further underlined by the recent demonstration of elevated T-cell related cytokines years before disease onset (78).

## Author contributions

PS, CT, DM, and AJM wrote the manuscript. PS contributed the figures. The idea for this review was conceived by PS. All authors contributed to the article and approved the submitted version.

## Conflict of interest

The authors declare that the research was conducted in the absence of any commercial or financial relationships that could be construed as a potential conflict of interest.

## Publisher's note

All claims expressed in this article are solely those of the authors and do not necessarily represent those of their affiliated organizations, or those of the publisher, the editors and the reviewers. Any product that may be evaluated in this article, or claim that may be made by its manufacturer, is not guaranteed or endorsed by the publisher.
